# Self-Expanding Versus Balloon Expanding Coronary Stents in Intervention of the Degenerated Saphenous Vein Graft: Memmingen Coronary Artery Bypass Stenosis Trial (MECAST)

**DOI:** 10.1155/2023/9412132

**Published:** 2023-04-06

**Authors:** Marcus Siry, Burak Duymaz, Simon Biesenberger, Deborah Siry, Vanessa Kammerer, Andreas E. May

**Affiliations:** ^1^Medizinische Klinik I, Klinikum Memmingen, Memmingen, Germany; ^2^University of Heidelberg, Department of Cardiology, Angiology and Pneumology, Heidelberg, Germany

## Abstract

**Objectives:**

The aim of this retrospective analysis was to compare the patient outcome after interventional therapy of saphenous vein graft (SVG) stenoses in an all-comers population receiving either self-expanding drug-eluting stents (SExS) or balloon expanding drug-eluting stents (BExS).

**Background:**

The interventional therapy of degenerated SVGs remains challenging. Diameter variations of stenotic segments and friable plaques can lead to malapposition and distal embolization with increased major adverse cardiac event (MACE) rates.

**Methods:**

107 patients with a total of 130 SVG interventions were separated into two groups according to either SExS (*n* = 51) or BExS (*n* = 56) treatment. Primary endpoint was the MACE rate, which is defined as the composite of cardiac death, myocardial infarction (MI), target vessel (TVR), and target lesion revascularization (TLR) at 30 days and at one-year follow-up.

**Results:**

Both patient groups did not differ significantly regarding patient characteristics. The patient outcome was significantly better in the SExS patient group: the MACE rate at 30 days was 1/51 (2.0%) in group SExS vs. 7/56 (12.5%) in group BExS; *p* < 0.05. At one-year follow-up, the MACE rate remained significantly lower in the SExS group 8/51(15.7%) vs. 20/56 (35.7%) in the BExS group, *p* < 0.02. Additionally, cardiac death occurred significantly later within the SExS patient group compared to the BExS group (*p* < 0.05). A better overall outcome of patients with de novo SVG-stenosis compared to patients with previous CABG (coronary artery bypass graft) intervention was noted in both groups.

**Conclusion:**

Our findings demonstrate that SVG treatment with SExS is safe and provides clinical benefits by comparatively improving short and especially long-term patient outcomes.

## 1. Introduction

Coronary artery bypass graft (CABG) surgery, whilst declining, is still a frequent procedure used worldwide for the treatment of coronary artery diseases. Although guidelines recommend a full arterial revascularization, most patients still receive at least one or more saphenous vein grafts in daily routine practice [1, 2]. In patients undergoing CABG, the rate of venous graft failure by graft occlusion is around 50% after 10 years follow-up [3]. This venous graft disease differs from classical arteriosclerosis in coronary artery disease: saphenous vein grafts (SVGs) typically degenerate and form stenotic segments over years developing diffuse intima hyperplasia with minor calcification and more friable plaques [4]. In consequence, these stenoses in older SVGs are often located close to ecstatic venous segments, making it difficult to choose the adequate stent diameter during interventional treatment. Another issue is the risk of distal embolization during the procedure due to the high debris burden in friable plaques. This can lead to slow or no reflow phenomenon with the risk of periprocedural MI. The use of an embolic protection device has been recommended based on the data of the SAFER trial [5]. However, it was downgraded (class IIa) in current ESC/EACTS guidelines on myocardial revascularization due to conflicting results in recent observational trials [2, 6, 7] and has never been used frequently in daily practice [8]. Overall, the interventional therapy of degenerated SVGs remains challenging and major cardiac adverse event (MACE) rates in bypass interventions are higher, compared to interventions in native coronaries [8]. Therefore, interventional treatment of the bypassed native coronary artery should be favored if possible [2]. However, bypassed native coronary arteries are often calcified, or chronically totally occluded which results in other challenges especially in the acute coronary syndrome [9].

Another important aspect is the difficulty in selecting the adequate coronary device. Until now, it is still unclear whether drug-eluting stents (DES), which proved superior compared to bare metal stents (BMS) for native coronary interventions, have the same advantages in the treatment of SVG stenoses. Although DES performed better compared to BMS in the ISAR-CABG trial at one-year follow-up, after a longer period of observation (5 years), MACE rates in both groups did not differ anymore [10, 11]. In the DIVA trial, where patients received either DES or BMS, the outcome in both groups remained equal [12]. In a recent trial comparing DES versus BMS in patients undergoing SVG intervention, the long-term follow-up after 5 years revealed a significantly lower MACE rate within the DES patient group. However, the list of exclusion criteria was extensive (previous stent implantation in the target SVG, need for concomitant intervention in a native coronary artery, SVG <6 months old, need for oral anticoagulation, etc.) [9].

Self-expanding drug-eluting stents (SExS) may have some advantages over conventional balloon expandable drug-eluting stents (BExS) due to their unique design (tight stent struts, active outward force, soft expansion, full stent apposition) [13]. Its soft expansion might reduce the risk of SVG perforation due to oversize ballooning or oversized stent implantation [9]. Furthermore, SExS have shown a full stent apposition rate without significant malapposition [14, 15]. Malapposition itself is an independent risk factor for stent thrombosis, leading to worse outcome [16]. In SVG interventions malapposition can occur in up to 50% of all cases [17] Therefore, SExS might be especially useful in SVG interventions with its varying diameter and friable plaques. In its current generation, the SExS is mounted on a balloon with a splittable sheath. Its delivery works similarly to a BExS; however, the balloon inflation serves for splitting the sheath only. While retrieving the splitted sheath, a slight or moderate resistance due to friction must be overcome. To prevent a distal coronary perforation, especially in SVG interventions, the modified floating wire technique facilitating its delivery process has been recommended [18].

A single center observation of 42 patients treated with SExS in degenerated SVG stenoses demonstrated a low restenosis rate of 4.8% after a median follow up of 13.4 months, proving the feasibility of SExS use in daily routine practice, however no peer group was evaluated [19].

Therefore, the Memmingen Coronary Artery bypass Stenosis Trial (MECAST) was initiated to analyze the potential clinical benefit of SExS in comparison to BExS regarding patient outcome for intervention of degenerated SVGs in daily routine practice.

## 2. Methods

### 2.1. Patients and SVG-Intervention Procedure (Figure 1)

**Figure 2:**
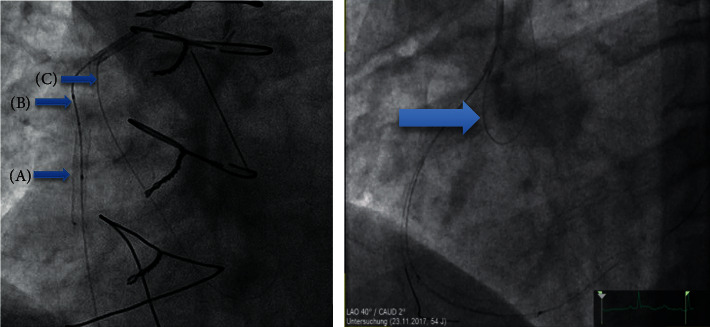
(a) SVG of RCA with stenosis located in proximal shaft: (b) modified floating wire technique. (a) SVG-stenosis located in proximal shaft, SExS (Xposition S® 3.0–3.5 ∗ 27 mm) implantation (A), sheath retrieval (B) with support of coronary wire (C). (b) Proximal SVG-RCA stenosis with implantation of Xposition S 3.0–3.5 ∗ 27 mm, sheath retrieval with insertion of an additional wire through the guiding catheter floating free in the ascending aorta => prevention of deep intubation with possible subsequent distal wire perforation. RCA = right coronary artery; SVG = saphenous vein graft; SExS: self-expanding drug eluting stent.

From Jan 2012 to Jan 2018, we performed 131 SVG interventions with DES stents in 108 patients at our institution. One patient receiving one procedure was lost to follow-up within the BExS group. An all-comers retrospective analysis was performed dividing these patients into two groups according to treatment (operator dependent): Group SExS mainly received (91.8%) second generation SExS (STENTYS-SES®; Xposition S®, nitinol alloy, sirolimus coated, cell area of 0.95 mm^2^, diameter range 2.5–4.5 mm, length 17–37 mm) (23) (patients: *N* = 51; SVG procedures: *N* = 61), while group BExS all received second generation BExS (diameter range 2.25–5.0 mm, length 8–32 mm) (patients: *N* = 56; SVG procedures: *N* = 69). The SExS stents were delivered through balloon inflation (12 atm) with a splittable sheath (Figure 2(a)) Due to higher resistance (splittable sheath) while retrieving the SExS delivery system, we modified the floating wire technique: an additional wire was inserted through the guiding catheter floating free in the ascending aorta and thereby preventing the guiding catheter from deep intubation and possible subsequent distal wire perforation (Figure 2(b)) [18–20]. Post-interventional treatment and antiplatelet therapy was performed according to current guidelines. All patients were followed up to a period of one-year either angiographically or by a phone call.

Patients who had received BMS, drug eluting balloon, or plain old balloon angioplasty (POBA) for SVG treatment were not included in the trial.

To analyze and quantify the plaque burden of the degenerated SVG, we used the SVG degeneration score analysis according to a predictive model [13].

The study was approved by the local ethics committee (Ethikkommision bei der Landesärztekammer Hessen, FF 125/2012) and previously registered (clinical registration number DE-ST2012-01; DEusches Sizing Register–DEUS).

All participants provided informed written consent.

### 2.2. Outcomes and Definitions

Technical success was defined as residual restenosis <20% of the target lesion and thrombolysis in myocardial infarction (TIMI) II-III flow at the end of the procedure.

Primary endpoints were the MACE rate within 30 days and at one-year follow-up. MACE was defined as a composite of target vessel revascularization (TVR), target lesion revascularization (TLR), myocardial infarction (MI) (according to current ESC guidelines [14]), and cardiac death. Additionally, stent thrombosis rate, defined according to the recommendations of the Academic Research Consortium, was analyzed [15].

### 2.3. Statistical Analysis

For all statistical analyses, the computer programs Excel (Microsoft, Redmond, USA), SPSS (Version 24, IBM, Armonk, USA) and MedCalc (Version 19.2, MedCalc Software, Ostend, Belgium) were used. Continuous data were summarized as mean ± standard deviation (normally distributed data) or median and interquartile range and compared using *t*-test or Mann–Whitney U test, as appropriate. Categorical variables were expressed as frequencies or percentages and were compared by using Fisher's exact test. Clinical event rate during follow up was calculated using the Kaplan–Meier curve and compared using the log-rank test. Hazard ratios were evaluated using Cox regression analysis. Results were considered significant with a *p* value of <0.05.

## 3. Results

### 3.1. Patients and Lesion Characteristics

**Figure 3:**
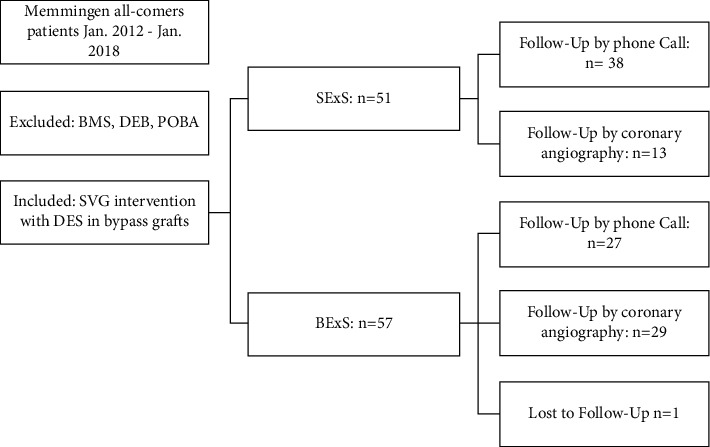
Retrospective data analysis. SVG = saphenous vein graft; SExS = self-expanding drug eluting stent; BExS = balloon expanding drug eluting stent; BMS = bare metal stent; DEB = drug eluting balloon; and POBA = plane old balloon angioplasty.

Patient recruitment details are depicted in Figure 3. Baseline characteristics are shown in Table 1. Overall, patient characteristics did not differ significantly between both patient groups. Most patients were male (SExS: 82.4%; BExS: 87.5%, *p*=0.46). The incidence of comorbidities was frequent in both groups (Diabetes, SExS: 49.0% vs. BExS: 42.9%, *p*=0.51; renal insufficiency (Creatinine clearance <90 mg/mmol), SExS: 51.0% vs. BExS: 37.5%, *p*=0.18; Peripheral artery occlusive disease, SExS: 33.3% vs. BExS: 33.9%, *p*=0.95). Nearly, half of the patients had acute coronary syndrome (SExS: 41.2% vs. BExS: 50.0%, *p*=0.36). The median age of the treated SVG was high (SExS: 17.0 vs. BExS: 16.8 years; *p*=0.49). The number of SVGs with reintervention was moderate (SExS: 21.6% vs. BExS: 25.0% *p*=0.62).

All patient and lesion characteristics were not normally distributed according to the Kolmogorov–Smirnov test. Therefore, a Mann–Whitney *U* test was applied which revealed no significant difference between both patient groups regarding baseline characteristics rendering them comparable.

Table 2 depicts procedural characteristics. In most patients, the SVGs were minor or moderately degenerated (≤50% of total SVG length), leading to a degeneration score of either 0 or 1, with no significant differences between both groups (group SExS: 77.0%; group BExS 63.8%, *p*=0.30).

Overall, 1.4 stents per procedure in both groups, *p*=0.99 were implanted. The usage of an embolic protection device was low (SExS: 8.2% vs. BExS: 11.6%, *p*=0.52). There was a significant difference in lesion preparation. According to user guidelines for SExS utilization in SVG/coronary lesions, pre- and post-dilatation is strongly recommended. Consequently, the pre-dilatation rate was significantly higher in the SExS group (SExS: 100% vs. BExS: 73.9%, *p* < 0.0001). Similarly, the post-dilatation rate differed significantly in both groups (SExS: 75.4%; BExS: 29.0%, *p* < 0.0001).

### 3.2. Outcomes

There was no significant difference between the SExS and BExS patient group regarding procedural success, which was defined as TIMI II or TIMI III flow (SExS: 100% vs BExS: 98.6%, *p*=0.32).

No periprocedural complications occurred within the BExS group. Of note, one patient died due to pericardial tamponade related to wire perforation of a distal coronary artery within the SExS group. This complication was related to retrieval of the SExS stent delivery system. To prevent further SExS-related perforations, we applied the modified floating wire technique [18, 19]. No similar additional complications occurred.

**Figure 4:**
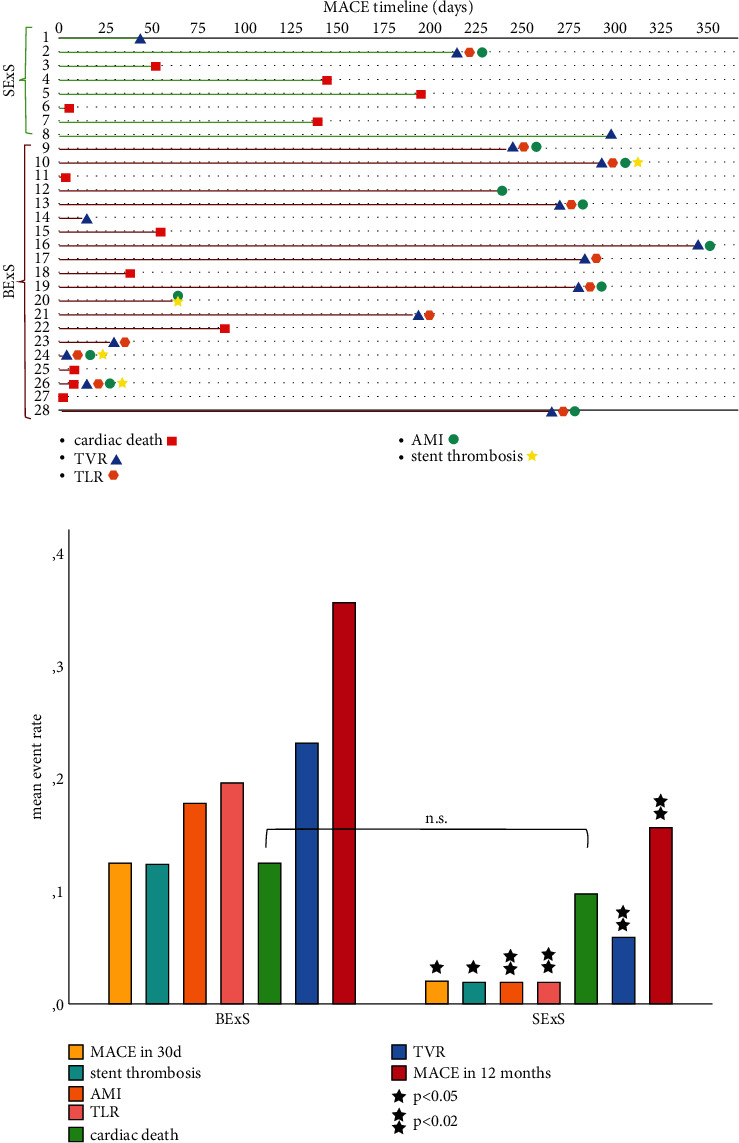
(a) MACE timeline of SExS and BExS patient groups. MACE = major adverse cardiac event; SExS = self-expanding drug eluting stent; BExS = balloon expanding drug eluting stent; TVR = target vessel revascularization; TLR = target lesion revascularization; AMI = acute myocardial infarction. (b) Mean event rates in BExS patient group compared to SExS group. SExS = self-expanding drug eluting stent; BExS = balloon expanding drug eluting stent; MACE = major adverse cardiac event; AMI = acute myocardial infarction; TLR = target lesion revascularization; TVR = target vessel revascularization.

Patient outcomes including the MACE rate are demonstrated in Table 3 and Figures 4(a) and 4(b). MACE at 30 days was lower in group SExS (2.0% vs. 12.5%, *p* < 0.05). At one-year follow-up, the MACE rate remained significantly lower in group SExS (15.7% vs. 35.7%, *p* < 0.02). Of note, one patient suffered from MI in the SExS group in comparison to 10 patients in group BExS (2.0% vs. 17.9%, *p* < 0.02). One patient in the SExS group underwent TLR in contrast to 11 patients in the BExS group (2.0% vs. 19.6%, *p* < 0.005). The number of TVR was also significantly lower in the SExS group (5.9% vs. 23.2%, *p* < 0.02).

Additionally, one stent thrombosis was noted within the SExS group in comparison to 7 stent thromboses in the BExS group (2.0% vs. 12.5%, *p* < 0.05).

**Figure 5:**
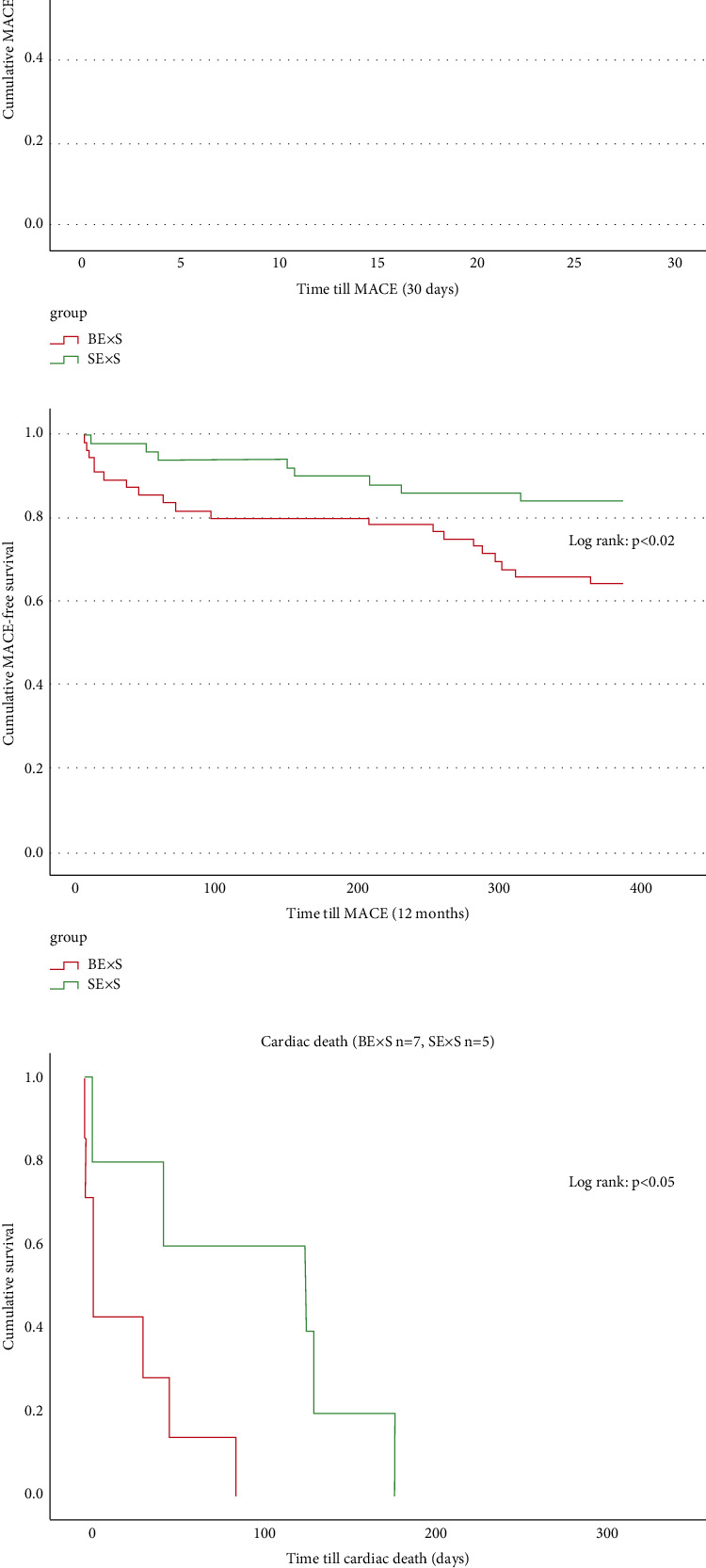
(a) Kaplan–Meier curve for cumulative MACE-free survival within 30 days in BExS and SExS patient groups (log rank *p* < 0.05). SExS = self-expanding drug eluting stent; BExS = balloon expanding drug eluting stent. (b) Central illustration: Kaplan–Meier curve for cumulative MACE-free survival within one year in BExS and SExS patient groups (log rank *p* < 0.02). SExS = self-expanding drug eluting stent; BExS = balloon expanding drug eluting stent. (c) Kaplan–Meier curve for cardiac death within 12 months in the BExS and SExS patient groups (log rank *p* < 0.05). SExS = self-expanding drug eluting stent; BExS = balloon expanding drug eluting stent.

The Kaplan–Meier curve analysis regarding MACE rate after 30 days and at one-year follow up demonstrated the superiority of SExS treatment compared to BExS. MACE-free survival was significantly better in the SExS patient group (MACE 30 days Log rank: *p* < 0.05, MACE 1 year Log rank: *p* < 0.02; Figures 5(a) and 5(b)). Furthermore, patients who suffered cardiac death within one-year follow-up died significantly earlier when treated by BExS as compared to SExS (Log rank: *p* < 0.05; Figure 5(c)).

**Figure 6:**
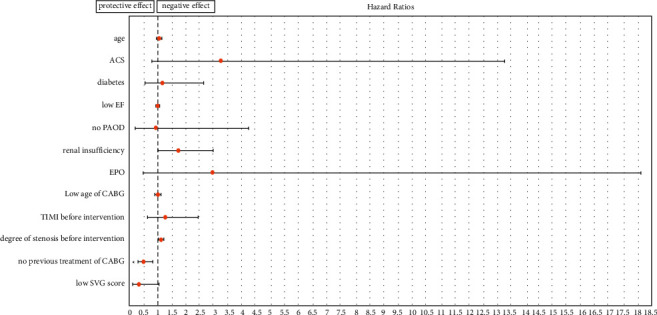
Forest plot depicting hazard ratios of several covariates. Statistical significance (*p* < 0.01) marked with a star. A hazard ratio <1 illustrates a protective effect on patient outcome, whereas a hazard ratio >1 represents a negative effect. ACS = acute coronary syndrome; EF = ejection fraction; PAOD = peripheral artery occlusive disease, EPD = embolic protection device; CABG = coronary artery bypass graft; TIMI = thrombolysis in myocardial infarction; and SVG = saphenous vein graft.

Cox regression analysis revealed several risk factor covariates with protective or negative effect on patient outcome (Figure 6). Of note, treatment of de novo SVG stenoses resulted in a significantly lower MACE rate in both patient groups (*p* < 0.01).

## 4. Discussion

In our trial, we performed a retrospective analysis of an all-comers patient population in the clinic of Memmingen over a period of six years who received an SVG intervention either by SExS (*n* = 51) or BExS (*n* = 56) and compared patient outcome specifically regarding MACE rate and cumulative survival.

Both patient groups (SExS and BExS) did not differ significantly regarding baseline characteristics and were therefore comparable. Of note, patients treated by SExS had slightly more comorbidities (Table 1). For procedural characteristics, the only significant difference between patient groups concerned lesion preparation (pre-dilatation SExS: 100% vs. BExS: 73.9%, *p* < 0.0001; post-dilatation SExS: 75.4% vs. BExS: 29.0%, *p* < 0.0001). User guidelines for SExS utilization strongly recommend pre- and post-dilatation, which explains the higher dilatation rate in the SExS group. It is highly unlikely that the lower pre- and post-dilatation rate had a negative impact on the outcome of the BExS group. In order to prevent distal embolization even direct stenting has been proposed, which in contrast led to a better short and long-term outcome after a one-year follow up compared to conventional BExS intervention [16], and as shown in a post hoc analysis of the DIVA trial, was associated with a lower stent thrombosis rate and lower target vessel MI-rate [17].

MACE rates in our BExS group were higher than in previous studies such as the ISAR-CABG or BASKET-SAVAGE trial. However, patients presenting with a cardiogenic shock or previous stent implantation or patients under oral anticoagulation were excluded in these trials. In contrast, in our study, we performed an all-comers analysis with no patient exclusions. This may explain the higher MACE-rates regarding the BExS patient group [9, 10].

On the other hand, we were able to demonstrate that patients treated by SExS had a significantly lower MACE rate after 30 days follow up than patients treated by BExS (2.0% vs. 12.5%, *p* < 0.05). This was mainly driven by a lower incidence of MI, TLR and TVR. The use of an embolic protection device was relatively low (SExS: 9.8% vs. BExS: 11.6%, *p*=0.43). Our results are comparable to the ADEPT trial [21], analyzing the previous generations of STENTYS stents (STENTYS bare-metal vs. STENTYS paclitaxel-eluting) in SVG interventions. In this trial, a low MACE rate at 30 days follow-up could be demonstrated in both groups (3.7% vs. 6.7%), with a low incidence of EPD use (<20%) [21]. The early benefit of SExS use in our trial might be due to soft expansion and full stent apposition with less distal embolization as mentioned above.

The main finding of our study was that after one-year follow-up, the MACE rate was still significantly lower in the SExS group than in the BExS group (15.7% vs. 35.7%, *p* < 0.02), again mostly driven by a lower incidence of MI (2.0% vs. 17.9%, *p* < 0.02), TLR (2.0% vs. 19.6%, *p* < 0.005) and TVR (5.9% vs. 23.2%, *p* < 0.015). Furthermore, the stent thrombosis rate was lower in the SExS group as well (2.0% vs. 12.5%, *p* < 0.05). MI-, TLR-, and TVR rates in the SExS patient group were even lower than in larger randomized trials (ISAR-CABG : MI: 4.1%, TLR: 7.2%, TVR: 11.5%; DIVA trial: MI: 10.0%, TLR: 9.0%, TVR: 12.0%) [10, 11]. Results suggest a long-term benefit of SExS. Whether this is due to full stent apposition in SVG stenoses or the sirolimus coating needs to be further explored.

Another major finding of our study was the following: Although the number of cardiac deaths was similarly low in both groups (SExS: *n* = 5; BExS: *n* = 7, *p*=0.66), cardiac death occurred significantly earlier within the BExS patient group indicating a longer survival rate after SExS intervention (*p* < 0.05). This could impact the patient outcome and needs to be further investigated in larger trials.

Cox regression analysis of risk factor covariates revealed several risk factors with hazard ratios >1, as well as protective factors (hazard ratio <1), however only one was significant: history of previous CABG treatment resulted in a higher one-year MACE rate in both patient groups as compared to de-novo SVG intervention (*p* < 0.01). This finding is highly interesting and suggests that within this patient subgroup treatment of the bypassed native coronary artery should be preferred, if possible.

In summary, our results suggest a potential benefit of SExS treatment in SVG stenoses. SExS may have a positive impact on the patient outcome, in particular by increasing cumulative survival and lowering incidence of the MACE rate.

Several limitations need to be mentioned. Our trial was a retrospective analysis of a single center only. The number of patients included in both groups was relatively small, due to the low incidence of patients in need of SVG intervention. The pre- and post-dilatation rates were overall higher within the SExS patient group. We only performed the analysis over a period of one-year follow-up. Post-procedural cardiac markers were not regularly acquired. Of note, the SExS tested in our study is currently no longer available (STENTYS-SES®).

## 5. Conclusion

In conclusion, we could demonstrate that the interventional therapy of degenerated SVGs by SExS is safe, even in patients with ACS. The SExS showed a clinical benefit over BExS regarding MACE rate at 30-days as well as at one-year follow-up, in particular concerning the rate of MI, TLR, and TVR. Furthermore, cardiac death occurred significantly later after treatment with SExS. The history of previous SVG intervention was an independent risk factor in both groups. Our findings should be further investigated in larger multicentric randomized trials and might enable an alternative interventional strategy in the treatment of SVG stenoses.

## Figures and Tables

**Figure 1:**
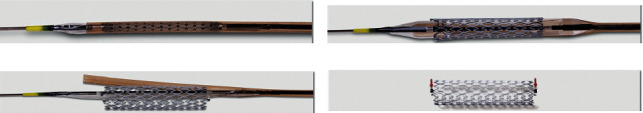
Delivery of self-expanding drug-eluting stent. (a) Stent is mounted on a semi-compliant balloon and is restrained by a splittable sheath. (b) Balloon inflation splits the sheath and releases the self-expanding stent. (c) Balloon is then deflated, leaving the 0.0032″ sheath between the stent and the vessel wall. (d) Balloon and sheath are then withdrawn leaving the stent opposed to the vessel wall. The two radiopaque stent markers are located at the edges of the stent (red arrows). With permission by STENTYS.

**Table 1:** Patient characteristics.

		SExS (*n* = 51)			BExS (*n* = 56)			*p* value
		*n *±SD	*n* (%)	Mean± SD	*n *±SD	*n* (%)	Mean± SD	
Sex	Female	9±2.87	17.6		7±2.56	12.5		0.458
	Male	42±5.05	82.4		49±5.15	87.5		
Age				76±1.21			74±1.16	0.360
ACS	Total	21±4.11	41.2		28±4.55	50.0		0.362
	STEMI	3±0.56	5.9		2±0.42	3.6		0.477
	NSTEMI	18±7.91	35.3		26±5.85	46.4		0.491
Diabetes	Medication	16±3.69	31.4		16±3.69	28.6		0.506
	Insulin	9±2.87	17.6		8±2.72	14.3		
EF				45±1.82			50±1.64	0.069
PAOD		17±3.87	33.3		19±3.95	33.9		0.948
Renal insufficiency	Low (creatinine clearance <90 mg/mmol)	18±3.87	35.3		15±3.59	26.8		0.181
	Moderate (creatinine clearance <60 mg/mmol)	6±2.38	11.8		3±1.71	5.4		
	Severe (creatinine clearance <30 mg/mmol)	1±1.00	2.0		0	0		
	Dialysis	1±1.00	2.0		3±1.71	5.4		
Age of CABG				17.0±0.80			16 .8±1.04	0.490
History of previous CABG intervention		11±1.96	21.6		14±2.38	25.0		0.616

Values are number (*n*)±standard deviation (SD), number (%) or mean±standard deviation. A *p* value <0.05 is considered significant. SExS = self-expanding drug eluting stent; BExS = balloon expanding drug eluting stent; ACS = acute coronary syndrome; EF = ejection fraction; PAOD = peripheral artery occlusive disease; CABG = coronary artery bypass graft.

**Table 2:** Procedural characteristics.

		SExS (procedures = 61)		BExS (procedures = 69)		*p* value
		*n *±SD	*n* (%)	*n *±SD	*n* (%)	
DES	First generation	5	8.2	0	0	0.516
	Second generation	56	91.8	69	100.0	
Procedures per patient		1.2±0.39		1.2±0.43		0.999
Stents per procedure		1.4±0.44		1.4±0.29		0.999
EPD		5±2.19	8.2	8±2.74	11.6	0.521
Lesion preparation	Pre-dilatation	61±5.69	100	51±5.57	73.9	<0.0001
	Post-dilatation	46±5.45	75.4	20±4.11	29.0	<0.0001
Lesion location	Ostial	13±3.42	21.3	18±3.49	26.1	0.462
	Medial	45±5.41	73.8	37±5.14	53.6	
	Distal	3±1.71	4.9	11±3.17	15.9	
	Multiple	0	0	3±1	4.3	
Low SVG score (0-1)		48±5.50	77.0	46±5.45	63.8	0.30
TIMI pre-procedure	0	3±1.71	4.9	5±2.19	7.2	0.979
	1	3±1.71	4.9	0	0	
	2	7±2.57	11.5	10±3.04	14.5	
	3	48±5.50	78.7	54±5.62	78.3	
TIMI post-procedure	0	0	0	1±1.00	1.4	0.742
	1	0	0	0	0	
	2	2±1.40	3.3	2±1.40	2.9	
	3	59±5.68	96.7	66±5.70	95.7	
Procedural success		61	100	68	98.6	0.317
Stent systems		**Overall 85 (1.7 per patient) implanted stents;** STENTYS® (paclitaxel), STENTYS-SES® (sirolimus), Xposition S® (sirolimus) Diameter range 2.5–4.5 mm Length 17–37 mm		**Overall 96 (1.8 per patient) implanted stents;** XIENCE®, promus element®, resolute integrety®, resolute Onyx®, Osiro®, BioMatrix® Diameter range 2.25–5.0 mm Length 8–32 mm		

Values are number (*n*)±standard deviation (SD), number (%) or mean±standard deviation. A *p* value <0.05 is considered significant. SExS = self-expanding drug eluting stent; BExS = balloon expanding drug eluting stent; DES = drug eluting stent; EPD = embolic protection device; SVG = saphenous vein graft; TIMI = thrombolysis in myocardial infarction.

**Table 3:** Outcome.

	SExS (*n* = 51)		BExS (*n* = 56)		*p* value
	*n *±SD	*n* (%)	*n *±SD	*n* (%)	
MACE 30 days	1±1.00	2.0	7±2.56	12.5	0.039
MACE 12 months	8±2.72	15.7	20±4.03	35.7	0.019
Cardiac death	5±2.18	9.8	7±2.56	12.5	0.660
MI	1±1.00	2.0	10±3.01	17.9	0.007
TLR	1±1.00	2.0	11±3.14	19.6	0.004
TVR	3±1.71	5.9	13±3.38	23.2	0.012
Stent thrombosis (possible + definite)	1±1.00	2.0	7±2.56	12.5	0.039

Values are number (*n*)±standard deviation (SD) or number (%). A *p* value<0.05 is considered significant. SExS = self-expanding drug eluting stent; BExS = balloon expanding drug eluting stent; MACE = major adverse cardiac event; MI = myocardial infarction; TLR = target lesion revascularization; and TVR = target vessel revascularization.

## Data Availability

The data used to support the findings of this study are available upon request from the corresponding author.
